# When time slows down: temporal distortion and addictive social media use

**DOI:** 10.3389/fpsyg.2025.1648583

**Published:** 2025-12-03

**Authors:** Leonarda Anna Vinci, Lucrezia Savioni, Stefano Triberti

**Affiliations:** Department of Psychology and Health Sciences, Pegaso University, Milan, Italy

**Keywords:** perception time, time distortion, temporal bias, time online, social media networking, addiction, internet use

## Abstract

This article represents a theoretical contribution on the involvement factors involved in temporal processing related to the use of social media networking. Current literature agrees in highlighting that individuals are not very accurate in making temporal judgments with respect to the duration and progression of events, since they often tend to perceive time in an accelerated or slowed way. The temporal distortion between real time intervals and subjective perceived ones involves several cognitive and emotional biases, implicated in the modalities and frequency of use of social media. These factors, associated with particular individual predispositions, can favor the development of a problematic use of social media in terms of temporal planning and overlapping of activities. This work discusses a possible cyclical model that broadens the understanding and emphasizes the central role of temporal distortion in the onset of social media addiction.

## 1 Introduction

The great development of social networks and their use has led to concerns over the risk of addiction (Bozzola et al., 2022; Kuss and Griffiths, 2017). Many studies have identified the important variables for classifying a person who uses social media in an addictive fashion (Zhao et al., 2022; Duradoni et al., 2020; Griffiths and Kuss, 2017). However, some studies have highlighted that the time-of-use is not really indicative of addiction (Charlton and Danforth, 2010; Milani et al., 2018; Triberti et al., 2018). Indeed many tend to spend a lot of time on social media due to work or other activities (e.g., social media managers, influencers), but without developing an addiction. Addiction develops when the subject progressively replaces their daily activities, such as study, work, etc., with consumption (Griffiths, 2005). That considered, the role of time-of-use in technology-related addiction deserves thorough analysis: it is possible to derive relevant information for assessment from time-related aspects, beyond the mere consideration of absolute time-of-use.

## 2 The perception of time

In literature, it is difficult to find a unified definition regarding the nature of time and its different applications. Jaspers (1913), similarly to Bergson (1889), distinguished “experience” from “knowledge” of time. The first refers to the measurable and objective aspects (the temporal scansion marked by clocks and calendars), while the second refers to subjective estimates.

Although we do not possess a “sense of time” (Wittmann, 2009), and therefore a receptor organ, such as the ear, we experience the passage of time through its indirect consequences, namely progressions or regressions. The literature has identified four temporal processes on which our judgments are based: (1) duration; (2) simultaneity and temporal order; (3) sensation of the passage of time; (4) mental time travel (Hinault et al., 2023). These are assessed through the use of personal time scales (e.g., the passing of the years) or more externalized scales, such as the change of season (Stojić et al., 2023) or measurable and objective scales.

Perceived time is positively related to: (1) perceptual vividness (more intense and complex stimuli are perceived to last longer), (2) ease of extracting information from the stimulus, (3) attention allocation (focusing attention on a stimulus extends its perceived duration), and (4) familiarity (novel events seem longer than familiar ones due to higher cognitive processing) (Matthews and Meck, 2016).

The perception of time is defined as a personal processing of temporal information through which individuals create short- and long-term behavioral and motivational plans (Seginer and Lens, 2015). Temporal perception is based on two aspects: succession, i.e., the recognition of the sequence of events, and duration, i.e., the estimation of the time interval between two events or the duration of a specific event (Fraisse, 1984).

Temporal perception allows us to confer coherence and linearity to the succession of events (Bausenhart et al., 2014; Vroomen and Keetels 2010); it is modulated both by external factors, such as complexity and degree of involvement (Block et al., 2010) and internal factors such as cognitive abilities (Coull et al., 2011; Fontes et al., 2016), emotional states (Droit-Volet et al. 2013; Ma et al., 2021; Micillo et al., 2024), personality (Lehockey et al., 2018; Wittmann and Paulus, 2008). motivations (Gable and Poole, 2012; Gupta, 2022), and stress (Hancock and Weaver, 2005).

These influence temporal judgments and become relevant in relation to the potentially problematic use of social media.

## 3 The role of time in social network addiction

Social networking sites (SNS) are “virtual communities where users can create individual public profiles, interact with friends, and meet others based on common interests” (Kuss and Griffiths, 2011).

Global Internet use has led to concerns, especially regarding young people, on the subject of addiction to technology and social networks. According to Griffiths’ (2005) addiction model, signs of social networking addiction include: mood alteration, salience (behavioral, cognitive and emotional preoccupation with SNS use), tolerance (increase in SNS use over time), withdrawal (unpleasant physical and emotional symptoms when SNS use is limited or stopped), conflict (interpersonal and intrapsychic problems) and rapid relapse after a period of abstinence. SN addiction makes individuals overly preoccupied, strongly motivated to stay online, distressed when unable to connect, and prone to organize activities around internet use (La Barbera et al., 2009).

Most studies have focused on how often and for how long users access social media each day. However, few studies have concretely classified the behavioral standards of SNS addiction, such as using it four times a day, for more than 60 min, or for more than 5 h a day (Hong et al., 2014). Two individuals who spend the same amount of time on SNS may or may not exhibit problematic behavior depending on other intervening factors. The objective and total time spent on SNS, taken alone, is not a reliable indicator of addiction (Orben and Przybylski, 2019). SNS addiction is defined as excessive social media use that impairs social functioning and/or psychological health and well-being (Andreassen and Pallesen 2014; Elphinston and Noller, 2011). However, meta-analysis found a weak correlation between time spent on SNS and psychological well-being, in terms of self-esteem, life satisfaction, loneliness, and depression (Huang, 2017). Time distortions, or an overestimation (upward time distortion) or underestimation (downward time distortion) of the duration of an activity or event, are common among technology-dependent individuals. We explore this phenomenon in relation to time spent on social media, an indicator of excessive use (Turel and Cavagnaro, 2019; Paasche et al., 2019). At-risk individuals exhibit altered perceptions of time, even in tasks unrelated to social media (Turel et al., 2018), suggesting the usefulness of analyzing the gap between perceived and actual time.

### 3.1 Gap between real and self-reported time internet use

Several studies have attempted to measure the discrepancy between the objective time of access to SNS and the subjective time experienced by subjects.

Although experimental settings offer a high level of stimulus control, they often lack ecological validity and generalization of the results (Matthews and Meck, 2014; Parry et al., 2021; van Rijn, 2018). Time spent on Facebook and number of logins are differentially related to outcomes (Junco, 2012a, 2012b). This suggests that self-reported and recorded measures may actually refer to distinct constructs, and that self-reports alone may not assess actual usage (Andrews et al., 2015; Vanden Abeele et al., 2013). Frequent smartphone checking habits and using a social app that can trigger access to other platforms increase overall usage time. Overestimation judgments have also been found in the estimation of short videos on Tik Tok and Instagram (Yang et al., 2024; Jiang et al., 2025). This suggests that temporal distortion may be characteristic of social media use. To better assess temporal bias, future studies should adopt multi-method approaches. Ecological Momentary Assessment (EMA) captures real-time perceptions of time use in natural settings, minimizing recall bias (Shiffman et al., 2008; Ahn et al., 2025; Minich et al., 2024). EMA has been effectively applied to problematic smartphone and social media use, especially among adolescents and university students, providing ecologically valid data on daily fluctuations in mood, behavior, and time perception (Coyne et al., 2022; Donisi et al., 2024). Passive smartphone and app logs offer objective benchmarks that, compared with self-reports, help quantify perceptual distortions. Prospective timing tasks can complement these methods by assessing temporal processing under controlled conditions, though with lower ecological validity.

Self-reported judgments alone would lead to uncertain measures and false positives because they are subject to various factors such as sleep, personality traits (Feldman et al., 2011; Walsh et al., 2010) and social desirability, i.e., users overestimate behaviors judged positive by their peer group to present themselves at their best (Araujo et al., 2017). Conversely, methods with higher ecological validity, such as EMA or passive sensing, offer greater insight into everyday behaviors but may present challenges in terms of implementation and participant compliance (Trull and Ebner-Priemer, 2013; Myin-Germeys et al., 2018).

The problem is not the time spent, but it’s ineffective management, which takes resources away from other priority activities (Katsitadze et al., 2025). Understanding the factors that influence the perception of time is crucial, especially when it is altered in addictions.

### 3.2 Time bias in social networks: use and addiction

The symptoms of problematic social media use are similar to those of other addictions, namely conflict, salience, withdrawal, relapse, tolerance, and mood alteration (Andreassen et al., 2012).

The perception of time is a subjective variable and strongly influenced by both the characteristics of the stimulus itself and those of the context in which it occurs (Droit-Volet and Meck, 2007; Lake et al., 2016): for example, time passes faster when we are in situations that are particularly salient/important for ourselves or when we are fully engaged in an activity, e.g., flow states (Rutrecht et al., 2021), while it can pass more slowly when we are just waiting (Wittmann, 2015; Mioni et al., 2023). The perception of time is often intertwined with our expectations (Shi et al., 2013); if I expect the train to arrive in 10 min, but it’s late, every extra minute will seem longer and longer combined with our restlessness. On the contrary, if I receive a message about the delay, expectations change and time seems to flow normally. These situations are typical of everyday life, generally transitory and context-related (Pariyadath and Eagleman, 2012; Karaaslan and Shi, 2024). However, in addiction contexts, they can take on specific characteristics.

Temporal biases in subjects with digital addiction are systematic (Cervigón-Carrasco et al., 2023) and also manifest themselves outside of compulsive use (Turel et al., 2018).

The meta-analysis by Gu et al. (2024) showed that non-risk users show temporal accuracy, greater projection towards the future and better awareness of the present.

Of interest is the “emotional distortion of time” (Lake et al., 2016), i.e., the impact that emotional stimuli have on time. The meta-analysis by Cui et al. (2023) has shown that the relevant factor is the level of arousal rather than the emotional valence (positive/negative). Highly activated emotions, positive or negative, tend to cause an overestimation of time, while low-activation emotions generate an underestimation. In behavioral addictions, such as SNS addiction, emotional arousal is often amplified by states of craving (e.g., from notifications, likes, viral content), impulsivity and affective dysregulation, which can interfere with a sense of time.

Literature shows that people prone to SNS addiction present some significant positive predictors including:

- a fatalistic present and negative past temporal orientation (Miceli et al., 2021);
- two forms of temporal distortion: see above;
- high impulsivity and impaired working memory (Wittmann, 2009; Moreira et al., 2016; Wegmann et al., 2020; Zhao et al., 2022): impulsive subjects overestimate time intervals and, consequently, undervalue the value of deferred rewards more than self-controlled individuals, especially in intense emotional conditions.

These behavioral and cognitive patterns fit within hyperbolic discounting models (Ainslie, 1975; Mazur, 1987), which explain the preference for immediate rewards over delayed ones. Ray and Bossaerts (2011) show that positive temporal dependence in the biological clock compresses future time perception, causing an exaggerated undervaluation of delayed rewards. This mechanism may amplify impulsive choices in SNS addiction, making immediate digital stimuli more compelling. Integrating this concept strengthens the explanatory power of hyperbolic discounting models by linking time perception distortions to impulsive social media use.

Dysfunctional metacognitive beliefs about the uncontrollability and danger of one’s thoughts predict addiction (Casale et al., 2021). In addition to awareness, the ability to control matters, consistent with compensatory use models: Internet regulates negative emotional states, but excessive use causes stress and discomfort. Time appears as slowed down by anticipatory stress linked to craving or emotional discomfort caused by the perception of abuse (Caplan, 2010).

It is important to consider moderating factors that influence temporal distortions, e.g., age: younger individuals tend to have more fragmented smartphone use, which may worsen difficulties in estimating time (Twenge et al., 2019). Socioeconomic status (SES) also plays a role, as different access to devices, digital literacy, and routines can affect usage patterns and time perception (Odgers and Jensen, 2020). Additionally, platform-specific features such as infinite scroll and autoplay (e.g., TikTok, Instagram Reels) can increase temporal distortions by promoting immersive use, unlike more functional platforms like LinkedIn. Recognizing these factors is crucial for identifying populations vulnerable to negative outcomes related to distorted time perception and problematic use.

### 3.3 A possible integrated model

**Figure 1:**
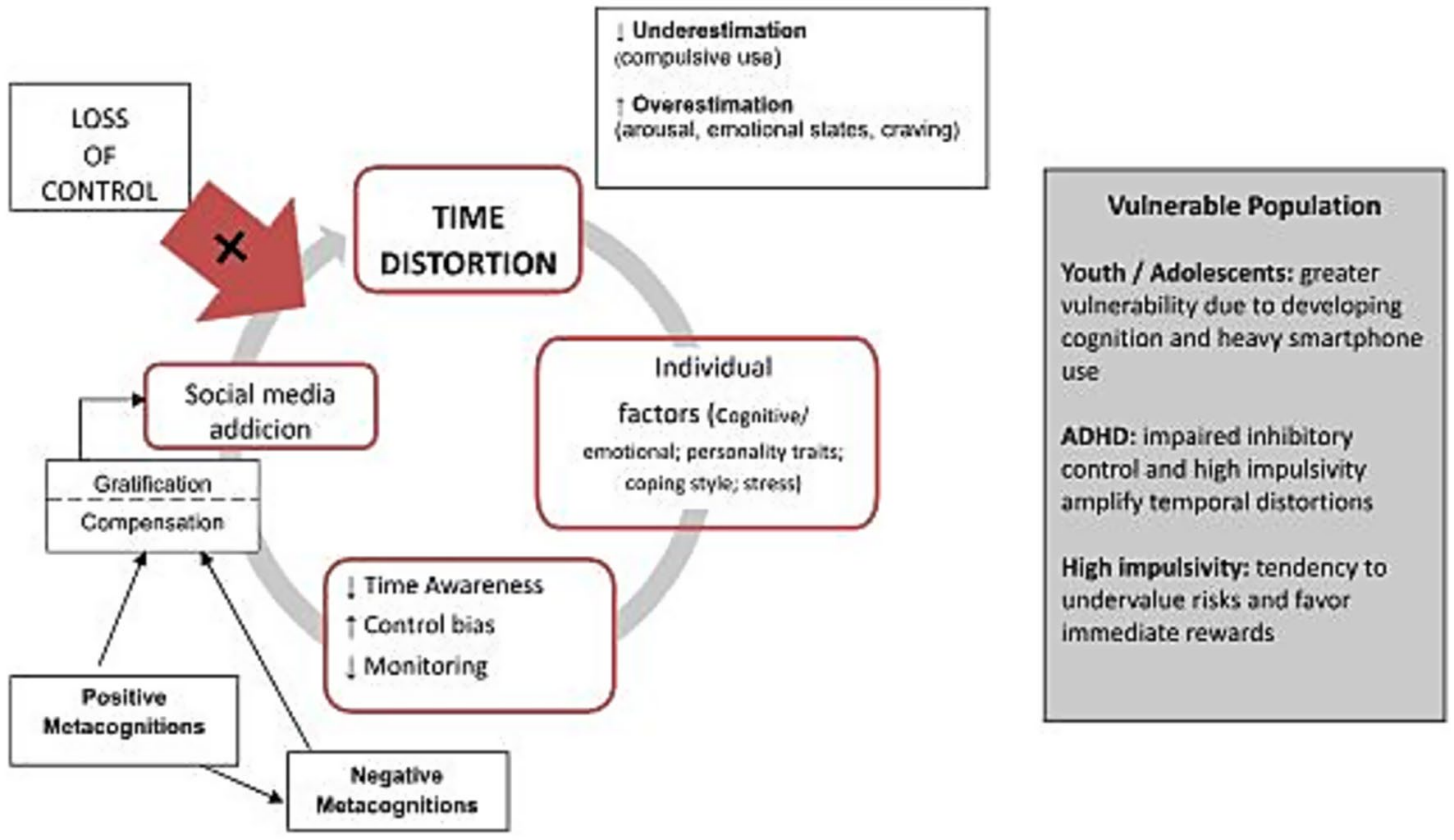
The main process that influence the social media addiction.

The literature has often focused on usage time as a variable determining addiction. However, we have seen how temporal bias alone is not sufficient, but is clinically relevant in the presence of vulnerabilities. Our hypothesis is that pre-existing biases in temporal representations may act as a contributing factor to the development of sensitivity to addictive conducts. In other words, people who have issues in representing time may be at higher risk of enacting behaviors that will become chronic due to other essential mechanisms. For example, an individual who does not realize the passage of time and/or overestimate time intervals between substance intake instances may be prone to develop addictive behaviors faster. While there is certainly evidence of time distortions in individuals with addiction or even impulsivity (as a pre-existent personality tendency) (Moreira et al., 2016; Wittmann et al., 2007), longitudinal data are needed to properly assess the contribution of this factor in the onset of addiction. Temporal distortion can interact with dysfunctional metacognitive control processes in a self-reinforcing cycle that fuels problematic technology use. Specifically, we hypothesized an integrated model (Figure 1) that identifies temporal distortion as a basic vulnerability, alongside executive, attentional and motivational dysfunctions that are implicated both in the dysregulation of digital behavior and in difficulties in temporal monitoring.

Social media use is supported by habitual and intermittent reinforcements (likes, notifications, followers), which amplify the perception of reward (Clark and Zack, 2023; Dores et al., 2025; Wang and Zhang, 2023). Platform’s design (progress bars, push notifications, personalisation) has been shown to further enhance engagement (Alutaybi et al., 2019; Montag and Elhai, 2023), along with external personal, professional and social demands, such as the use of LinkedIn tags to showcase work accomplishments (Weisi and Hajizadeh, 2025; Nor et al., 2025).

However, these are often cited as a cause of excessive use, not everyone develops addiction: some remain regular users or low-risk, without impairments in daily functioning. Positive reinforcement explains the onset, but not sustained use or loss of control.

In accordance with theories of internal vulnerability to time perception (Wittmann and Paulus, 2008), temporal distortion could serve as a marker that helps distinguish individuals with or without addiction.

The model hypothesises that individuals at greatest risk of addiction exhibit a temporal perception deficit, distorting the duration of events and experiencing frustration. The intensity of distortion is modulated by characteristics such as poor emotional regulation, impulsivity, low tolerance of boredom and frustration, anxiety, perfectionism and a temporal perspective focused on the negative present and past with limited orientation towards the future (Miceli et al., 2021).

These factors interact with socio-digital dynamics to act as ‘cognitive slot machines’ (Voinea et al., 2024), maximising engagement and pursuit of immediate rewards in line with the hyperbolic discounting mechanism. Impaired temporal awareness reduces the ability to self-monitor behavior and promotes maladaptive coping strategies, such as excessive social media use (S-REF model; Wells and Matthews, 1996).

Using social media after experiencing momentary gratification, relief, and increased reactivity to stimuli reinforces positive metacognitive beliefs about the usefulness of social media for internal emotional and cognitive regulation (“TikTok videos relax me”). This increases excitability and the desire to use social media (craving) and, at the same time, reinforces negative metacognitions regarding the uncontrollability of thoughts (“If I ignore notifications, I’ll feel worse”; “I cannot stop even though I know I’ve wasted a lot of time”). This results in weakened self-regulation efforts and increase in compulsive, uncontrollable behaviors (Meynadier et al., 2025; Spada et al., 2008), potentially leading to digital abstinence. Ultimately, a cycle of maintenance can develop, in which the loss of control intensifies time distortion. This progressively compromises decision-making processes and goal-oriented activities in daily life.

## 4 Conclusion

As SNS become a habitual part of our daily lives, concerns about their potential negative effects on physical and mental well-being are growing. This article explored the subjective perception of time associated with the use of SNS. Based on the review of current literature, we emphasized that the factor “usage time” is not a dependent variable and directly related to abuse or addiction. Users are not aware of the time they spend online on a daily basis (Horwood and Anglim, 2019; Schmuck, 2020) and most spend more than 2 h per day on the web. Objective measurement of time spent on individual devices is neither indicative of abuse nor does it provide information on subjective distress and functional impairment.

Intense use for work, education and routine purposes differs from compulsive use. Online time becomes relevant when it takes away significant time from other daily activities (Triberti et al., 2018). That considered, it may be tempting to assume that usage time is not problematic; at the same time, it becomes even more important to identify problematic activities.

Time distortion, considered as a constant, is an indicator that can still help diagnosis. We propose an integrated cyclical model that hypothesizes a pre-existing temporal distortion as a contributing factor to sensitivity to addictive conducts.

The hypothesis supports and could enrich the I-PACE model (Interaction of Person-Affect-Cognition-Execution; Brand et al., 2019) in which addiction is given by the interaction between individual predisposing factors, affective, cognitive and executive.

One strength of our hypothesis is that it could give clear indications for falsification. For example, there is sparse evidence of temporal distortions in populations that are highly engaged in an activity (e.g., social media, video games) (Jiang et al., 2025; Burén et al., 2023; Wood and Griffiths, 2007), yet without developing a risk for addiction. Alternatively, most studies compare high- and low-risk groups for addiction, where problematic use is already present. Further exploration of similar results may drive the design of experiments on more specific types, frequency or correlates of temporal distortions, to demonstrate whether and under what conditions they could act as contributory factors in the development of addiction, or not.

The strength of this hypothesis lies in the possibility of testing it through quantitative research and psychological interventions. Indeed, future research could focus on longitudinal studies to identify causal relations between pre-existing temporal distortions and vulnerability to addiction. Clinicians could also develop interventions (e.g., randomized controlled trials) where resources are given to addicted patients to improve their temporal representations vs. control groups, to assess the effects on mental health issues. This, in fact, could also translate into the possibility of systematically inserting standardized measures of temporal perception in the assessment of SNS abuse. To test hypotheses and to facilitate improved self-regulation of time, the following may be helpful: cognitive-behavioral techniques, such as implementing limits on usage and offline time; mindfulness to increase awareness of the present moment; using apps to self-monitor time and emotions. On the other hand, one limitation is that we did not consider the perceived temporal discrepancy in overall Internet/social media use, combining it comprehensively with all the biases that could influence the process. Future research could focus on possible differences between overall Internet and social media use, as well as between platforms, in terms of frequency, quantity, and age of users.

## Data Availability

The original contributions presented in the study are included in the article/supplementary material, further inquiries can be directed to the corresponding author.
